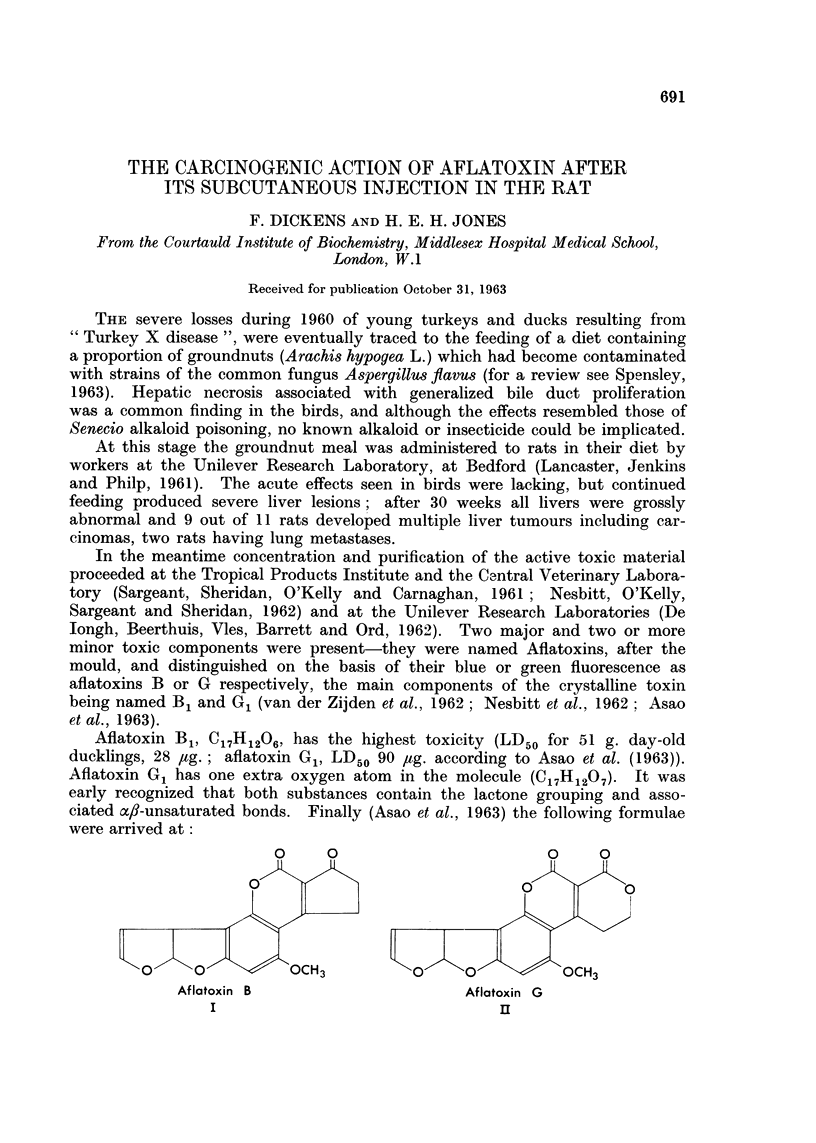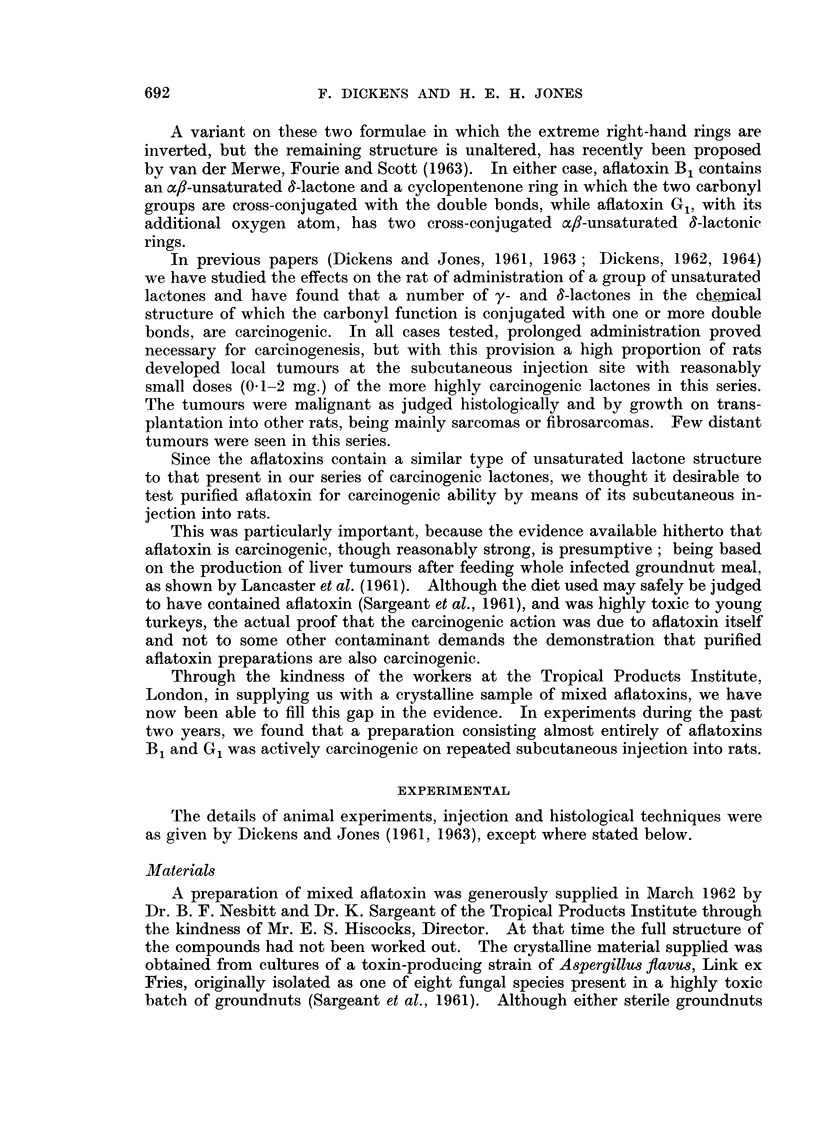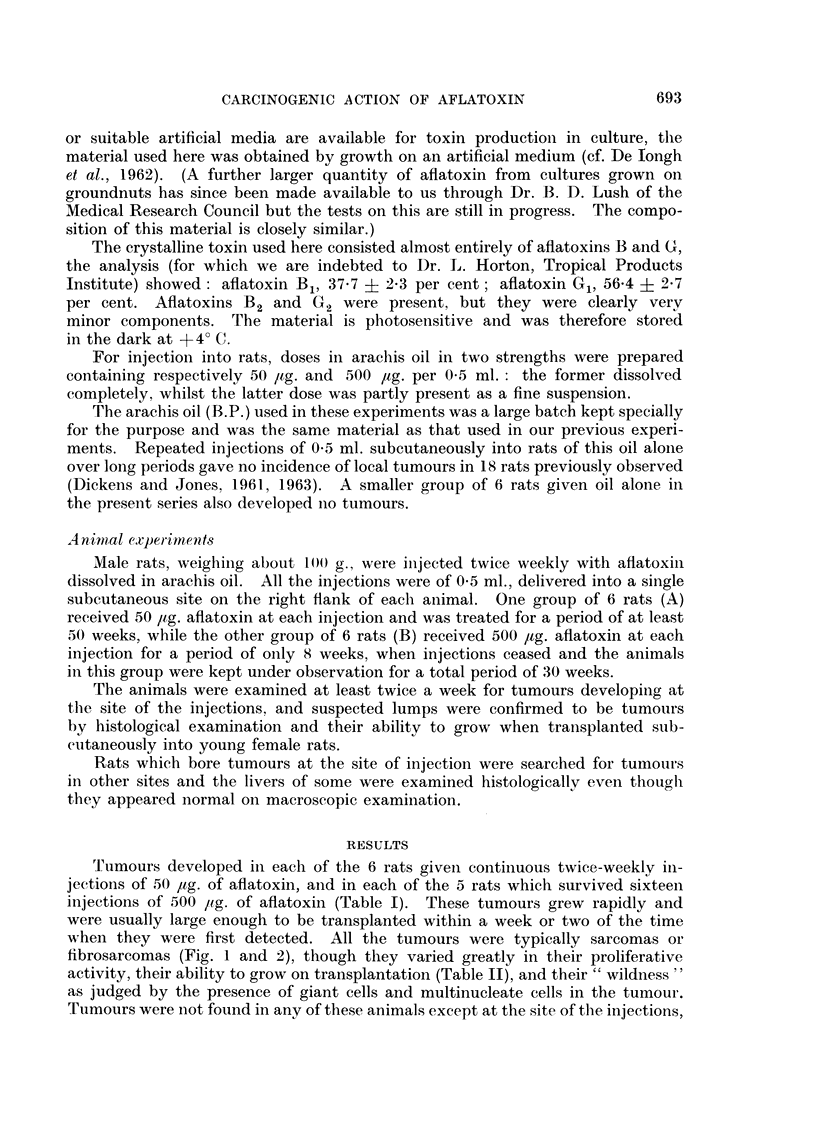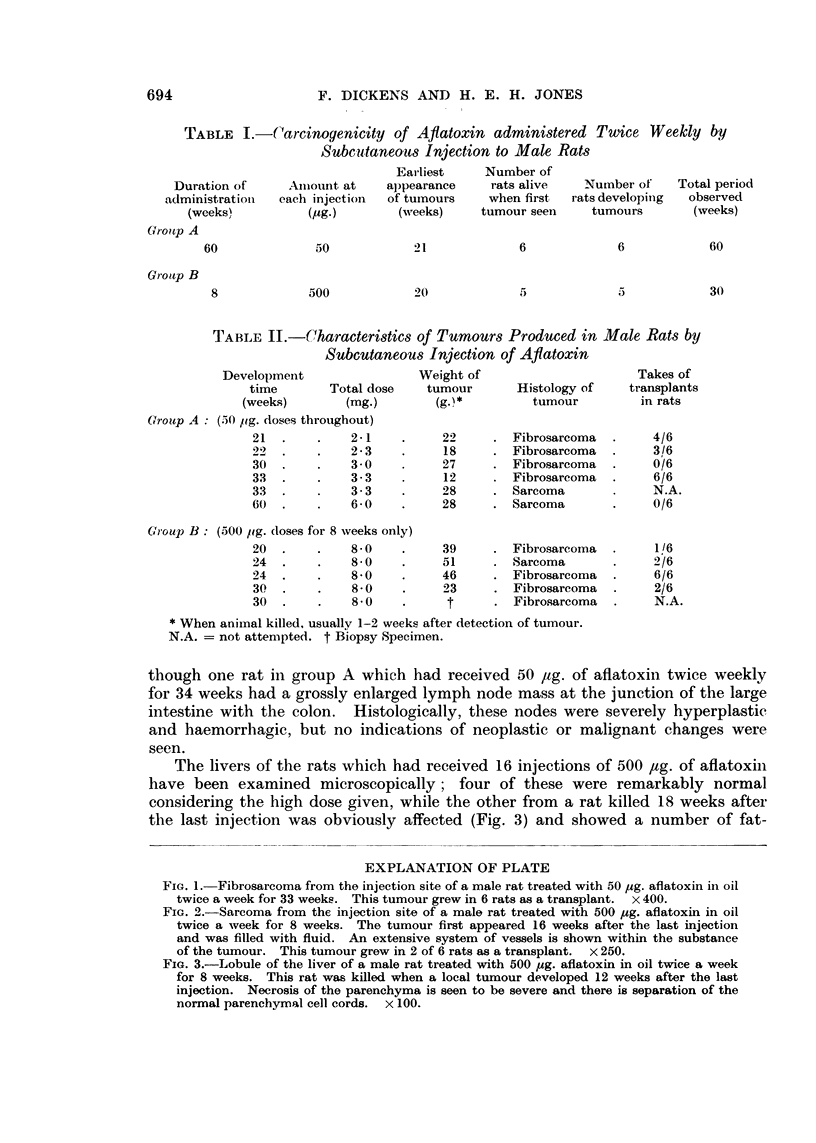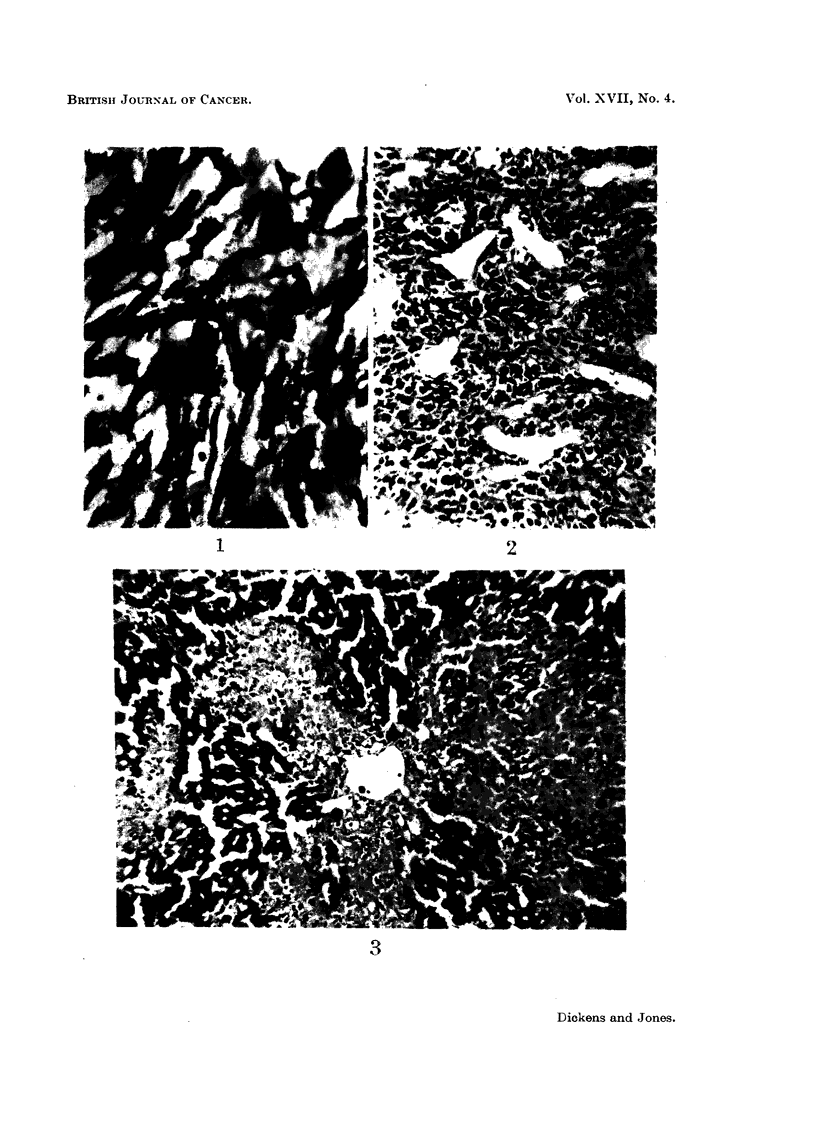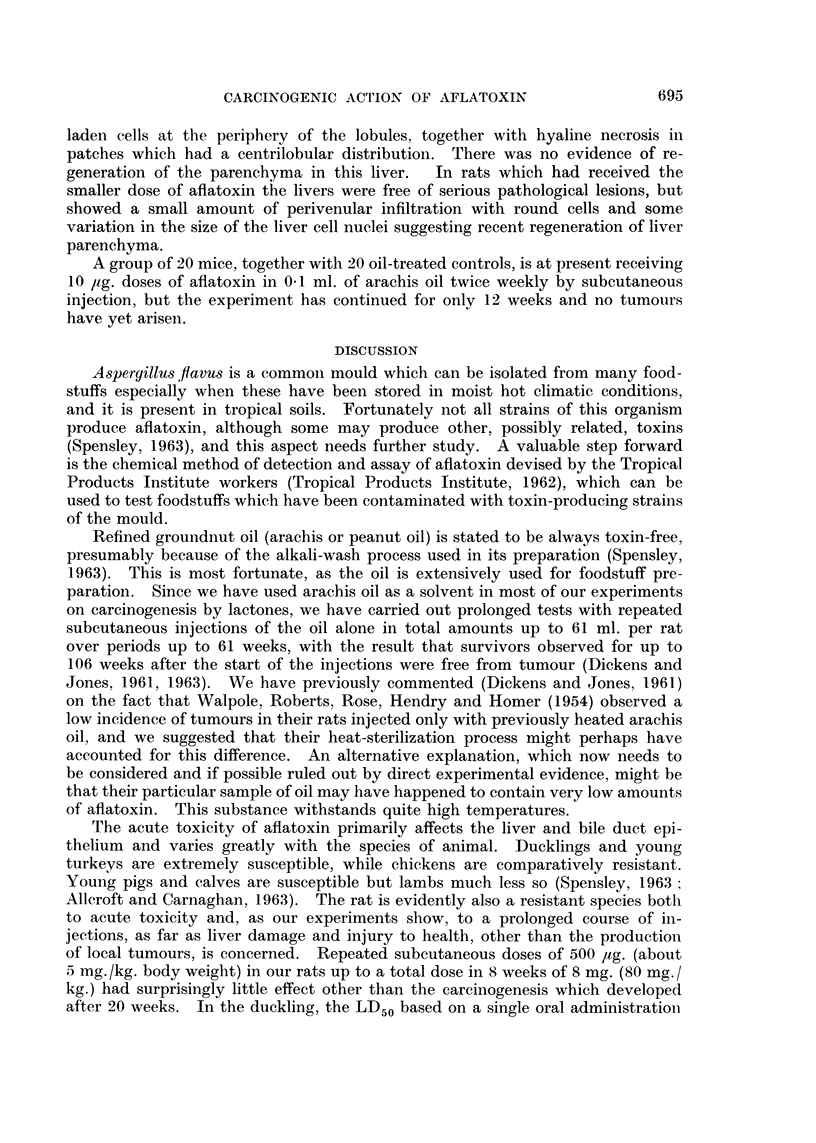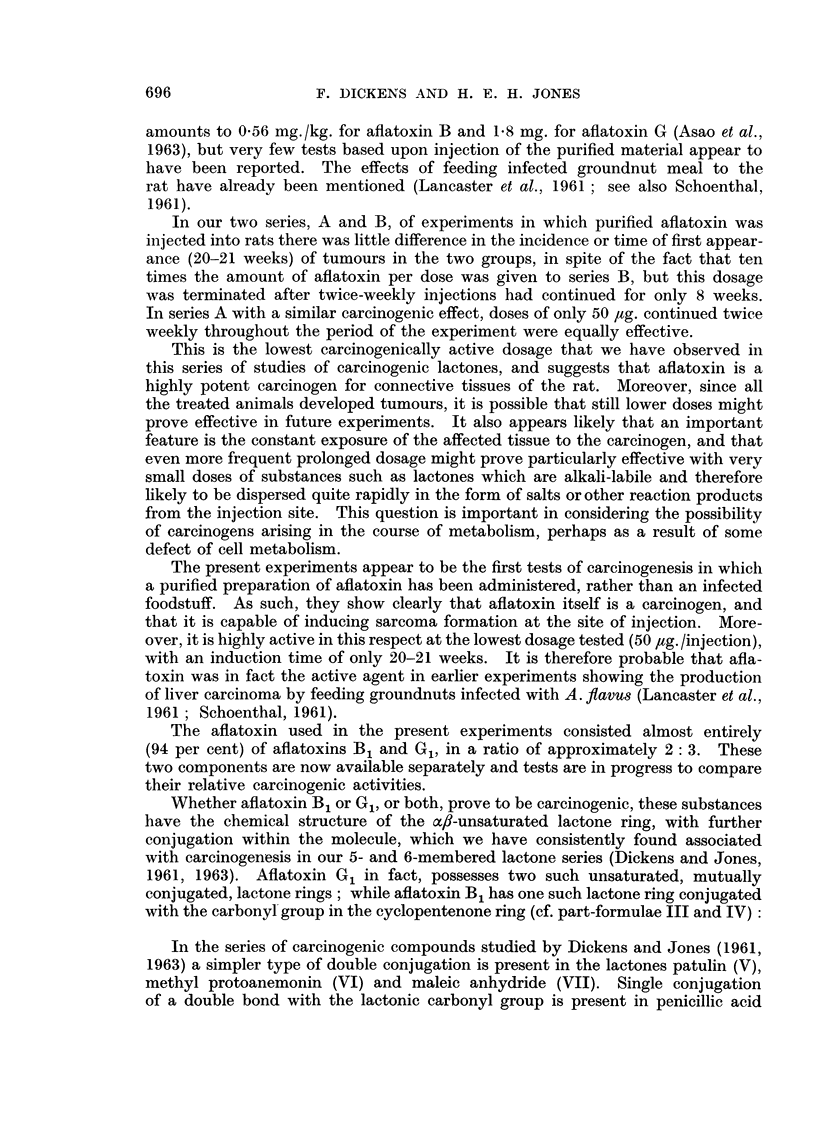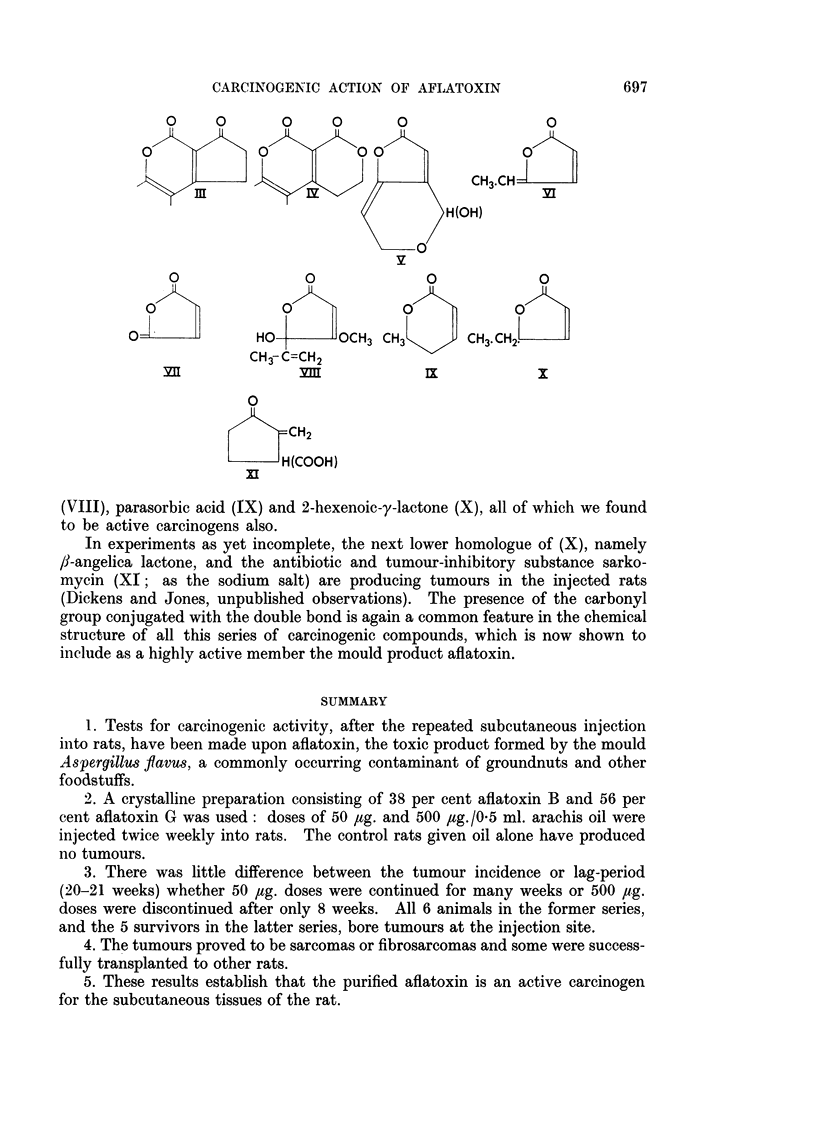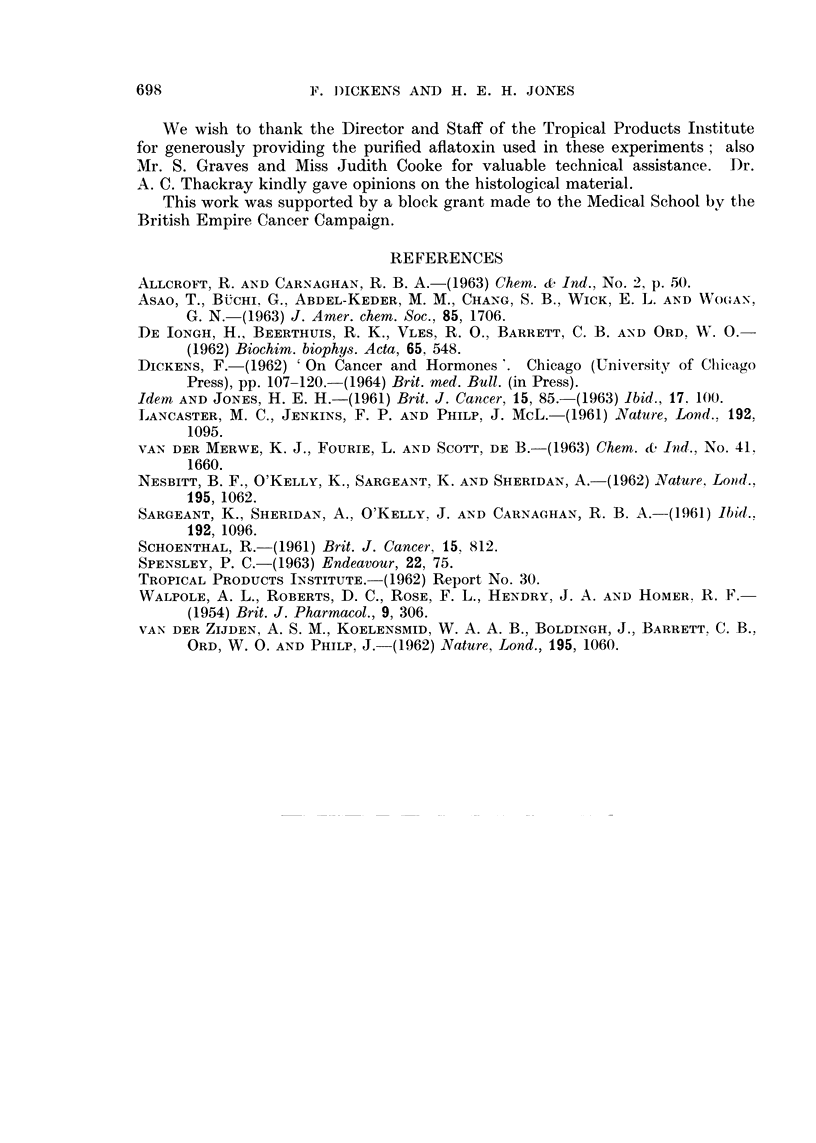# The Carcinogenic Action of Aflatoxin after its Subcutaneous Injection in the Rat

**DOI:** 10.1038/bjc.1963.88

**Published:** 1963-12

**Authors:** F. Dickens, H. E. H. Jones

## Abstract

**Images:**


### THE CARCINOGENIC ACTION OF AFLATOXIN AFTER ITS SUBCUTANEOUS INJECTION IN THE RAT

F. DICKENS AND H. E. H. JONES

*From the Courtauld Institute of Biochemistry, Middlesex Hospital Medical School, London, W.1*

Received for publication October 31, 1963

THE severe losses during 1960 of young turkeys and ducks resulting from " Turkey X disease ", were eventually traced to the feeding of a diet containing a proportion of groundnuts (*Arachis hypogea* L.) which had become contaminated with strains of the common fungus *Aspergillus flavus* (for a review see Spensley, 1963). Hepatic necrosis associated with generalized bile duct proliferation was a common finding in the birds, and although the effects resembled those of *Senecio* alkaloid poisoning, no known alkaloid or insecticide could be implicated.

At this stage the groundnut meal was administered to rats in their diet by workers at the Unilever Research Laboratory, at Bedford (Lancaster, Jenkins and Philp, 1961). The acute effects seen in birds were lacking, but continued feeding produced severe liver lesions ; after 30 weeks all livers were grossly abnormal and 9 out of 11 rats developed multiple liver tumours including carcinomas, two rats having lung metastases.

In the meantime concentration and purification of the active toxic material proceeded at the Tropical Products Institute and the Central Veterinary Laboratory (Sargeant, Sheridan, O'Kelly and Carnaghan, 1961 ; Nesbitt, O'Kelly, Sargeant and Sheridan, 1962) and at the Unilever Research Laboratories (De Iongh, Beerthuis, Vles, Barrett and Ord, 1962). Two major and two or more minor toxic components were present—they were named Aflatoxins, after the mould, and distinguished on the basis of their blue or green fluorescence as aflatoxins B or G respectively, the main components of the crystalline toxin being named $B_1$ and $G_1$ (van der Zijden *et al.*, 1962 ; Nesbitt *et al.*, 1962 ; Asao *et al.*, 1963).

Aflatoxin $B_1$, $C_{17}H_{12}O_6$, has the highest toxicity ($LD_{50}$ for 51 g. day-old ducklings, 28 $\mu$g. ; aflatoxin $G_1$, $LD_{50}$ 90 $\mu$g. according to Asao *et al.* (1963)). Aflatoxin $G_1$ has one extra oxygen atom in the molecule ($C_{17}H_{12}O_7$). It was early recognized that both substances contain the lactone grouping and associated $\alpha\beta$-unsaturated bonds. Finally (Asao *et al.*, 1963) the following formulae were arrived at :

Aflatoxin B
I

Aflatoxin G

II

A variant on these two formulae in which the extreme right-hand rings are inverted, but the remaining structure is unaltered, has recently been proposed by van der Merwe, Fourie and Scott (1963). In either case, aflatoxin $B_1$ contains an $\alpha\beta$-unsaturated $\delta$-lactone and a cyclopentenone ring in which the two carbonyl groups are cross-conjugated with the double bonds, while aflatoxin $G_1$, with its additional oxygen atom, has two cross-conjugated $\alpha\beta$-unsaturated $\delta$-lactonic rings.

In previous papers (Dickens and Jones, 1961, 1963; Dickens, 1962, 1964) we have studied the effects on the rat of administration of a group of unsaturated lactones and have found that a number of $\gamma$- and $\delta$-lactones in the chemical structure of which the carbonyl function is conjugated with one or more double bonds, are carcinogenic. In all cases tested, prolonged administration proved necessary for carcinogenesis, but with this provision a high proportion of rats developed local tumours at the subcutaneous injection site with reasonably small doses (0·1–2 mg.) of the more highly carcinogenic lactones in this series. The tumours were malignant as judged histologically and by growth on transplantation into other rats, being mainly sarcomas or fibrosarcomas. Few distant tumours were seen in this series.

Since the aflatoxins contain a similar type of unsaturated lactone structure to that present in our series of carcinogenic lactones, we thought it desirable to test purified aflatoxin for carcinogenic ability by means of its subcutaneous injection into rats.

This was particularly important, because the evidence available hitherto that aflatoxin is carcinogenic, though reasonably strong, is presumptive; being based on the production of liver tumours after feeding whole infected groundnut meal, as shown by Lancaster *et al.* (1961). Although the diet used may safely be judged to have contained aflatoxin (Sargeant *et al.*, 1961), and was highly toxic to young turkeys, the actual proof that the carcinogenic action was due to aflatoxin itself and not to some other contaminant demands the demonstration that purified aflatoxin preparations are also carcinogenic.

Through the kindness of the workers at the Tropical Products Institute, London, in supplying us with a crystalline sample of mixed aflatoxins, we have now been able to fill this gap in the evidence. In experiments during the past two years, we found that a preparation consisting almost entirely of aflatoxins $B_1$ and $G_1$ was actively carcinogenic on repeated subcutaneous injection into rats.

### EXPERIMENTAL

The details of animal experiments, injection and histological techniques were as given by Dickens and Jones (1961, 1963), except where stated below.

### *Materials*

A preparation of mixed aflatoxin was generously supplied in March 1962 by Dr. B. F. Nesbitt and Dr. K. Sargeant of the Tropical Products Institute through the kindness of Mr. E. S. Hiscocks, Director. At that time the full structure of the compounds had not been worked out. The crystalline material supplied was obtained from cultures of a toxin-producing strain of *Aspergillus flavus*, Link ex Fries, originally isolated as one of eight fungal species present in a highly toxic batch of groundnuts (Sargeant *et al.*, 1961). Although either sterile groundnuts or suitable artificial media are available for toxin production in culture, the material used here was obtained by growth on an artificial medium (cf. De Iongh *et al.*, 1962). (A further larger quantity of aflatoxin from cultures grown on groundnuts has since been made available to us through Dr. B. D. Lush of the Medical Research Council but the tests on this are still in progress. The composition of this material is closely similar.)

The crystalline toxin used here consisted almost entirely of aflatoxins B and G, the analysis (for which we are indebted to Dr. L. Horton, Tropical Products Institute) showed: aflatoxin $B_1$, 37·7 ± 2·3 per cent; aflatoxin $G_1$, 56·4 ± 2·7 per cent. Aflatoxins $B_2$ and $G_2$ were present, but they were clearly very minor components. The material is photosensitive and was therefore stored in the dark at +4° C.

For injection into rats, doses in arachis oil in two strengths were prepared containing respectively 50 μg. and 500 μg. per 0·5 ml.: the former dissolved completely, whilst the latter dose was partly present as a fine suspension.

The arachis oil (B.P.) used in these experiments was a large batch kept specially for the purpose and was the same material as that used in our previous experiments. Repeated injections of 0·5 ml. subcutaneously into rats of this oil alone over long periods gave no incidence of local tumours in 18 rats previously observed (Dickens and Jones, 1961, 1963). A smaller group of 6 rats given oil alone in the present series also developed no tumours.

*Animal experiments*

Male rats, weighing about 100 g., were injected twice weekly with aflatoxin dissolved in arachis oil. All the injections were of 0·5 ml., delivered into a single subcutaneous site on the right flank of each animal. One group of 6 rats (A) received 50 μg. aflatoxin at each injection and was treated for a period of at least 50 weeks, while the other group of 6 rats (B) received 500 μg. aflatoxin at each injection for a period of only 8 weeks, when injections ceased and the animals in this group were kept under observation for a total period of 30 weeks.

The animals were examined at least twice a week for tumours developing at the site of the injections, and suspected lumps were confirmed to be tumours by histological examination and their ability to grow when transplanted subcutaneously into young female rats.

Rats which bore tumours at the site of injection were searched for tumours in other sites and the livers of some were examined histologically even though they appeared normal on macroscopic examination.

### RESULTS

Tumours developed in each of the 6 rats given continuous twice-weekly injections of 50 μg. of aflatoxin, and in each of the 5 rats which survived sixteen injections of 500 μg. of aflatoxin (Table I). These tumours grew rapidly and were usually large enough to be transplanted within a week or two of the time when they were first detected. All the tumours were typically sarcomas or fibrosarcomas (Fig. 1 and 2), though they varied greatly in their proliferative activity, their ability to grow on transplantation (Table II), and their " wildness " as judged by the presence of giant cells and multinucleate cells in the tumour. Tumours were not found in any of these animals except at the site of the injections,

TABLE I.—*Carcinogenicity of Aflatoxin administered Twice Weekly by Subcutaneous Injection to Male Rats*

| Duration of administration (weeks) | Amount at each injection ($\mu$g.) | Earliest appearance of tumours (weeks) | Number of rats alive when first tumour seen | Number of rats developing tumours | Total period observed (weeks) |
|---|---|---|---|---|---|
| *Group A* | | | | | |
| 60 | 50 | 21 | 6 | 6 | 60 |
| *Group B* | | | | | |
| 8 | 500 | 20 | 5 | 5 | 30 |

TABLE II.—*Characteristics of Tumours Produced in Male Rats by Subcutaneous Injection of Aflatoxin*

| Development time (weeks) | Total dose (mg.) | Weight of tumour (g.)* | Histology of tumour | Takes of transplants in rats |
|---|---|---|---|---|
| *Group A* : (50 $\mu$g. doses throughout) | | | | |
| 21 | 2·1 | 22 | Fibrosarcoma | 4/6 |
| 22 | 2·3 | 18 | Fibrosarcoma | 3/6 |
| 30 | 3·0 | 27 | Fibrosarcoma | 0/6 |
| 33 | 3·3 | 12 | Fibrosarcoma | 6/6 |
| 33 | 3·3 | 28 | Sarcoma | N.A. |
| 60 | 6·0 | 28 | Sarcoma | 0/6 |
| *Group B* : (500 $\mu$g. doses for 8 weeks only) | | | | |
| 20 | 8·0 | 39 | Fibrosarcoma | 1/6 |
| 24 | 8·0 | 51 | Sarcoma | 2/6 |
| 24 | 8·0 | 46 | Fibrosarcoma | 6/6 |
| 30 | 8·0 | 23 | Fibrosarcoma | 2/6 |
| 30 | 8·0 | † | Fibrosarcoma | N.A. |

* When animal killed, usually 1–2 weeks after detection of tumour.
N.A. = not attempted. † Biopsy Specimen.

though one rat in group A which had received 50 $\mu$g. of aflatoxin twice weekly for 34 weeks had a grossly enlarged lymph node mass at the junction of the large intestine with the colon. Histologically, these nodes were severely hyperplastic and haemorrhagic, but no indications of neoplastic or malignant changes were seen.

The livers of the rats which had received 16 injections of 500 $\mu$g. of aflatoxin have been examined microscopically ; four of these were remarkably normal considering the high dose given, while the other from a rat killed 18 weeks after the last injection was obviously affected (Fig. 3) and showed a number of fat-

### EXPLANATION OF PLATE

FIG. 1.—Fibrosarcoma from the injection site of a male rat treated with 50 $\mu$g. aflatoxin in oil twice a week for 33 weeks. This tumour grew in 6 rats as a transplant. $\times 400$.

FIG. 2.—Sarcoma from the injection site of a male rat treated with 500 $\mu$g. aflatoxin in oil twice a week for 8 weeks. The tumour first appeared 16 weeks after the last injection and was filled with fluid. An extensive system of vessels is shown within the substance of the tumour. This tumour grew in 2 of 6 rats as a transplant. $\times 250$.

FIG. 3.—Lobule of the liver of a male rat treated with 500 $\mu$g. aflatoxin in oil twice a week for 8 weeks. This rat was killed when a local tumour developed 12 weeks after the last injection. Necrosis of the parenchyma is seen to be severe and there is separation of the normal parenchymal cell cords. $\times 100$.

1

2

3

Dickens and Jones.

laden cells at the periphery of the lobules, together with hyaline necrosis in patches which had a centrilobular distribution. There was no evidence of regeneration of the parenchyma in this liver. In rats which had received the smaller dose of aflatoxin the livers were free of serious pathological lesions, but showed a small amount of perivenular infiltration with round cells and some variation in the size of the liver cell nuclei suggesting recent regeneration of liver parenchyma.

A group of 20 mice, together with 20 oil-treated controls, is at present receiving 10 $\mu$g. doses of aflatoxin in 0·1 ml. of arachis oil twice weekly by subcutaneous injection, but the experiment has continued for only 12 weeks and no tumours have yet arisen.

### DISCUSSION

*Aspergillus flavus* is a common mould which can be isolated from many foodstuffs especially when these have been stored in moist hot climatic conditions, and it is present in tropical soils. Fortunately not all strains of this organism produce aflatoxin, although some may produce other, possibly related, toxins (Spensley, 1963), and this aspect needs further study. A valuable step forward is the chemical method of detection and assay of aflatoxin devised by the Tropical Products Institute workers (Tropical Products Institute, 1962), which can be used to test foodstuffs which have been contaminated with toxin-producing strains of the mould.

Refined groundnut oil (arachis or peanut oil) is stated to be always toxin-free, presumably because of the alkali-wash process used in its preparation (Spensley, 1963). This is most fortunate, as the oil is extensively used for foodstuff preparation. Since we have used arachis oil as a solvent in most of our experiments on carcinogenesis by lactones, we have carried out prolonged tests with repeated subcutaneous injections of the oil alone in total amounts up to 61 ml. per rat over periods up to 61 weeks, with the result that survivors observed for up to 106 weeks after the start of the injections were free from tumour (Dickens and Jones, 1961, 1963). We have previously commented (Dickens and Jones, 1961) on the fact that Walpole, Roberts, Rose, Hendry and Homer (1954) observed a low incidence of tumours in their rats injected only with previously heated arachis oil, and we suggested that their heat-sterilization process might perhaps have accounted for this difference. An alternative explanation, which now needs to be considered and if possible ruled out by direct experimental evidence, might be that their particular sample of oil may have happened to contain very low amounts of aflatoxin. This substance withstands quite high temperatures.

The acute toxicity of aflatoxin primarily affects the liver and bile duct epithelium and varies greatly with the species of animal. Ducklings and young turkeys are extremely susceptible, while chickens are comparatively resistant. Young pigs and calves are susceptible but lambs much less so (Spensley, 1963; Allcroft and Carnaghan, 1963). The rat is evidently also a resistant species both to acute toxicity and, as our experiments show, to a prolonged course of injections, as far as liver damage and injury to health, other than the production of local tumours, is concerned. Repeated subcutaneous doses of 500 $\mu$g. (about 5 mg./kg. body weight) in our rats up to a total dose in 8 weeks of 8 mg. (80 mg./kg.) had surprisingly little effect other than the carcinogenesis which developed after 20 weeks. In the duckling, the $LD_{50}$ based on a single oral administration amounts to 0·56 mg./kg. for aflatoxin B and 1·8 mg. for aflatoxin G (Asao *et al.*, 1963), but very few tests based upon injection of the purified material appear to have been reported. The effects of feeding infected groundnut meal to the rat have already been mentioned (Lancaster *et al.*, 1961 ; see also Schoenthal, 1961).

In our two series, A and B, of experiments in which purified aflatoxin was injected into rats there was little difference in the incidence or time of first appearance (20–21 weeks) of tumours in the two groups, in spite of the fact that ten times the amount of aflatoxin per dose was given to series B, but this dosage was terminated after twice-weekly injections had continued for only 8 weeks. In series A with a similar carcinogenic effect, doses of only 50 $\mu$g. continued twice weekly throughout the period of the experiment were equally effective.

This is the lowest carcinogenically active dosage that we have observed in this series of studies of carcinogenic lactones, and suggests that aflatoxin is a highly potent carcinogen for connective tissues of the rat. Moreover, since all the treated animals developed tumours, it is possible that still lower doses might prove effective in future experiments. It also appears likely that an important feature is the constant exposure of the affected tissue to the carcinogen, and that even more frequent prolonged dosage might prove particularly effective with very small doses of substances such as lactones which are alkali-labile and therefore likely to be dispersed quite rapidly in the form of salts or other reaction products from the injection site. This question is important in considering the possibility of carcinogens arising in the course of metabolism, perhaps as a result of some defect of cell metabolism.

The present experiments appear to be the first tests of carcinogenesis in which a purified preparation of aflatoxin has been administered, rather than an infected foodstuff. As such, they show clearly that aflatoxin itself is a carcinogen, and that it is capable of inducing sarcoma formation at the site of injection. Moreover, it is highly active in this respect at the lowest dosage tested (50 $\mu$g./injection), with an induction time of only 20–21 weeks. It is therefore probable that aflatoxin was in fact the active agent in earlier experiments showing the production of liver carcinoma by feeding groundnuts infected with *A. flavus* (Lancaster *et al.*, 1961 ; Schoenthal, 1961).

The aflatoxin used in the present experiments consisted almost entirely (94 per cent) of aflatoxins $B_1$ and $G_1$, in a ratio of approximately 2 : 3. These two components are now available separately and tests are in progress to compare their relative carcinogenic activities.

Whether aflatoxin $B_1$ or $G_1$, or both, prove to be carcinogenic, these substances have the chemical structure of the $\alpha\beta$-unsaturated lactone ring, with further conjugation within the molecule, which we have consistently found associated with carcinogenesis in our 5- and 6-membered lactone series (Dickens and Jones, 1961, 1963). Aflatoxin $G_1$ in fact, possesses two such unsaturated, mutually conjugated, lactone rings ; while aflatoxin $B_1$ has one such lactone ring conjugated with the carbonyl group in the cyclopentenone ring (cf. part-formulae III and IV) :

In the series of carcinogenic compounds studied by Dickens and Jones (1961, 1963) a simpler type of double conjugation is present in the lactones patulin (V), methyl protoanemonin (VI) and maleic anhydride (VII). Single conjugation of a double bond with the lactonic carbonyl group is present in penicillic acid

(VIII), parasorbic acid (IX) and 2-hexenoic-$\gamma$-lactone (X), all of which we found to be active carcinogens also.

In experiments as yet incomplete, the next lower homologue of (X), namely $\beta$-angelica lactone, and the antibiotic and tumour-inhibitory substance sarkomycin (XI; as the sodium salt) are producing tumours in the injected rats (Dickens and Jones, unpublished observations). The presence of the carbonyl group conjugated with the double bond is again a common feature in the chemical structure of all this series of carcinogenic compounds, which is now shown to include as a highly active member the mould product aflatoxin.

### SUMMARY

1. Tests for carcinogenic activity, after the repeated subcutaneous injection into rats, have been made upon aflatoxin, the toxic product formed by the mould *Aspergillus flavus*, a commonly occurring contaminant of groundnuts and other foodstuffs.

2. A crystalline preparation consisting of 38 per cent aflatoxin B and 56 per cent aflatoxin G was used: doses of 50 $\mu$g. and 500 $\mu$g./0·5 ml. arachis oil were injected twice weekly into rats. The control rats given oil alone have produced no tumours.

3. There was little difference between the tumour incidence or lag-period (20–21 weeks) whether 50 $\mu$g. doses were continued for many weeks or 500 $\mu$g. doses were discontinued after only 8 weeks. All 6 animals in the former series, and the 5 survivors in the latter series, bore tumours at the injection site.

4. The tumours proved to be sarcomas or fibrosarcomas and some were successfully transplanted to other rats.

5. These results establish that the purified aflatoxin is an active carcinogen for the subcutaneous tissues of the rat.

We wish to thank the Director and Staff of the Tropical Products Institute for generously providing the purified aflatoxin used in these experiments ; also Mr. S. Graves and Miss Judith Cooke for valuable technical assistance. Dr. A. C. Thackray kindly gave opinions on the histological material.

This work was supported by a block grant made to the Medical School by the British Empire Cancer Campaign.

### REFERENCES

ALLCROFT, R. AND CARNAGHAN, R. B. A.—(1963) *Chem. & Ind.*, No. 2, p. 50.

ASAO, T., BÜCHI, G., ABDEL-KEDER, M. M., CHANG, S. B., WICK, E. L. AND WOGAN, G. N.—(1963) *J. Amer. chem. Soc.*, **85**, 1706.

DE IONGH, H., BEERTHUIS, R. K., VLES, R. O., BARRETT, C. B. AND ORD, W. O.—(1962) *Biochim. biophys. Acta*, **65**, 548.

DICKENS, F.—(1962) 'On Cancer and Hormones'. Chicago (University of Chicago Press), pp. 107–120.—(1964) *Brit. med. Bull.* (in Press).

*Idem* AND JONES, H. E. H.—(1961) *Brit. J. Cancer*, **15**, 85.—(1963) *Ibid.*, **17**, 100.

LANCASTER, M. C., JENKINS, F. P. AND PHILP, J. McL.—(1961) *Nature, Lond.*, **192**, 1095.

VAN DER MERWE, K. J., FOURIE, L. AND SCOTT, DE B.—(1963) *Chem. & Ind.*, No. 41, 1660.

NESBITT, B. F., O'KELLY, K., SARGEANT, K. AND SHERIDAN, A.—(1962) *Nature, Lond.*, **195**, 1062.

SARGEANT, K., SHERIDAN, A., O'KELLY, J. AND CARNAGHAN, R. B. A.—(1961) *Ibid.*, **192**, 1096.

SCHOENTHAL, R.—(1961) *Brit. J. Cancer*, **15**, 812.

SPENSLEY, P. C.—(1963) *Endeavour*, **22**, 75.

TROPICAL PRODUCTS INSTITUTE.—(1962) Report No. 30.

WALPOLE, A. L., ROBERTS, D. C., ROSE, F. L., HENDRY, J. A. AND HOMER, R. F.—(1954) *Brit. J. Pharmacol.*, **9**, 306.

VAN DER ZIJDEN, A. S. M., KOELENSMID, W. A. A. B., BOLDINGH, J., BARRETT, C. B., ORD, W. O. AND PHILP, J.—(1962) *Nature, Lond.*, **195**, 1060.